# Viral Mimicry to Usurp Ubiquitin and SUMO Host Pathways

**DOI:** 10.3390/v7092849

**Published:** 2015-08-28

**Authors:** Peter Wimmer, Sabrina Schreiner

**Affiliations:** 1 Novartis Pharma Germany, Roonstrasse 25, 90429 Nürnberg, Germany; wimmerpeter@gmx.de; 2 Institute of Virology, Technische Universität München, Trogerstrasse 30, 81675 München, Germany; 3 Helmholtz Zentrum München, Ingolstädter Landstrasse 1, 85764 Neuherberg/München, Germany

**Keywords:** SUMO, ubiquitin, virus, analogue, mimicry, SENP, DUB

## Abstract

Posttranslational modifications (PTMs) of proteins include enzymatic changes by covalent addition of cellular regulatory determinants such as ubiquitin (Ub) and small ubiquitin-like modifier (SUMO) moieties. These modifications are widely used by eukaryotic cells to control the functional repertoire of proteins. Over the last decade, it became apparent that the repertoire of ubiquitiylation and SUMOylation regulating various biological functions is not restricted to eukaryotic cells, but is also a feature of human virus families, used to extensively exploit complex host-cell networks and homeostasis. Intriguingly, besides binding to host SUMO/Ub control proteins and interfering with the respective enzymatic cascade, many viral proteins mimic key regulatory factors to usurp this host machinery and promote efficient viral outcomes. Advanced detection methods and functional studies of ubiquitiylation and SUMOylation during virus-host interplay have revealed that human viruses have evolved a large arsenal of strategies to exploit these specific PTM processes. In this review, we highlight the known viral analogs orchestrating ubiquitin and SUMO conjugation events to subvert and utilize basic enzymatic pathways.

## 1. Introduction 

Ubiquitinylation and SUMOylation are reversible processes that regulate key factors. Consequently, these PTMs (posttranslational modification) contribute to signaling pathways such as gene regulation, epigenetics, differentiation, protein degradation, and tumorigenesis. Phosphorylation, acetylation, and methylation of substrates directly influence whether ubiquitin/SUMO (small ubiquitin-like modifier) moieties are conjugated/deconjugated to proteins, and how the ubiquitin/SUMO chains will be assembled [1,2,3]. Any of these processes can be altered by analogs of viral origin either circumventing or supporting ubiquitinylation/SUMOylation of host or viral substrates. Such events are often beneficial for virus progeny production and/or constitute immune evasion strategies associated with efficient viral gene expression and replication of the viral genome [4]. Therefore, identification and detailed understanding of virus-mediated PTM manipulation by viral analogs infiltrating ubiquitin/SUMO pathways will provide valuable information for future antiviral drug development and/or novel immunotherapies.

### 1.1. Ubiquitinylation and De-Ubiquitinylation Processes

Ubiquitinylation is an enzymatic cascade involving the covalent linkage of a small protein called ubiquitin to the amino group of a lysine residue in a substrate protein through an isopeptide bond to the C-terminal glycine of ubiquitin [5,6]. Beside canonically transferring ubiquitin to a lysine residue, non-canonically linked poly-ubiquitin chains can arise when ubiquitin is conjugated to the amine group in the N-terminus of the substrate protein [7]. 

**Figure 1:**
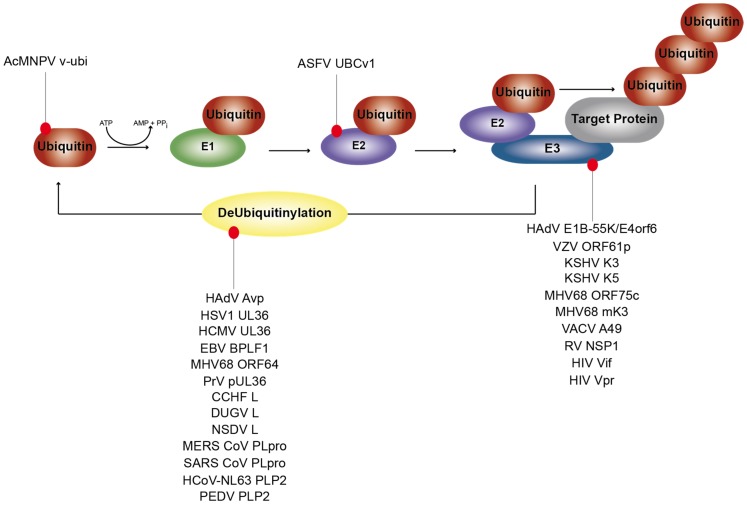
Overview of some viruses that modulate host ubiquitinylation pathways. Listed are viruses known to encode proteins exhibiting functions of cellular regulatory proteins involved in ubiquitinylation. Described in detail in the text.

Ubiquitin is a conserved, 76-amino-acid protein (8.5 kDa), containing seven lysine residues: Lys6, Lys11, Lys27, Lys29, Lys33, Lys48, and Lys63 [8]. Ubiquitin is activated by a factor called E1, a ubiquitin-activating enzyme (UBA1, UBA6) in an ATP-dependent manner, when a high-energy thioester bond is formed between the carboxy terminus of ubiquitin and an internal Cys residue of E1. Activated ubiquitin is then transferred and conjugated to the active site cysteine of one of many ubiquitin-conjugating enzymes, called E2. Finally, the ubiquitin ligase, called E3, catalyzes the formation of an isopeptide bond between the target protein and ubiquitin moieties (see Figure 1). In some cases, poly-ubiquitin chains can also be attached to mono-ubiquitinylated lysine residues [7]. 

Conjugation at Lys63 primarily mediates protein trafficking among subcellular compartments, whereas modification at Lys48 classically designates the substrate as a target for proteasomal degradation [9]. Three classes of E3 ubiquitin ligases are defined depending on their HECT domain, a Cullin-RING, or their U-box motif [10,11]. RBR (ring-between-ring) E3 ligases have also been reported, and these combine the structural features of HECT and RING E3 ligases [12]. 

De-ubiquitinylation is the reverse process of ubiquitinylation, where ubiquitin is cleaved from the target protein (see Figure 1), catalyzed by de-ubiquitinylation enzymes (DUBs) or ubiquitin-specific proteases (USPs) [13]. Such enzymes hydrolyze ubiquitin precursors directly after translation into free ubiquitin molecules, recycle ubiquitins that are conjugated on target proteins destined for proteasomal degradation, or modulate ubiquitin conjugation of target proteins. DUBs are involved in various biological processes including immune and inflammatory responses, chromatin remodeling, cellular antiviral signaling [14].

### 1.2. SUMOylation and De-SUMOylation Processes

**Figure 2:**
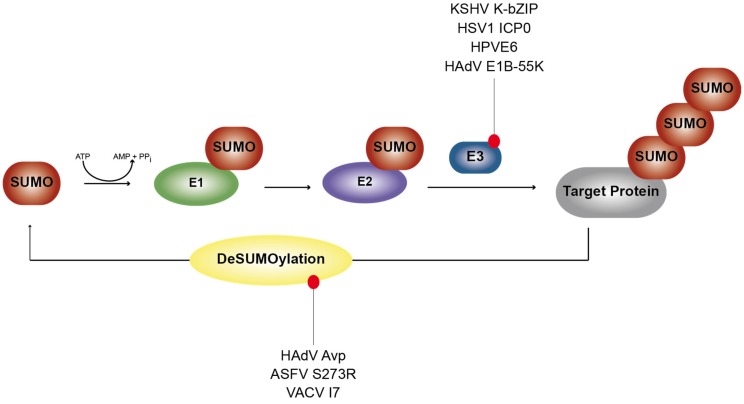
Overview of some viruses that modulate host SUMOylation pathways. Listed are viruses known to encode proteins exhibiting functions of cellular regulatory proteins involved in SUMOylation. Described in detail in the text.

SUMOylation is an enzymatic cascade involving the covalent linkage of a small ubiquitin-like modifier (SUMO) protein to a lysine residue of a substrate by a three-step enzymatic pathway. SUMO is activated by the E1 enzymes SAE1/SAE2 prior to being conjugated to the unique E2 enzyme Ubc9. Finally, different classes of a very few known SUMO E3 ligases, such as PIAS, RING-domain-containing and TRIM proteins, connect SUMOs to target proteins via an isopeptide bond formed between the C-terminal carboxyl group of SUMO and the amino group of the lysine residue in the substrate (see Figure 2) [4]. SENPs (SUMO-specific proteases) are required for the maturation of SUMO precursors and to reverse SUMOylation.

In contrast to ubiquitinylation, four different SUMO paralogs (~12 kDa) are known (SUMO-1, SUMO-2, SUMO-3, and SUMO-4), which regulate various cellular processes and functions such as gene regulation, cell differentiation, and tumor development [15]. In comparison to ubiquitin, much less is known about the functions that are modulated by SUMO proteins. The covalent attachment of SUMO to target proteins mainly depends on the target consensus motif ψKxE/D (where ψ represents a large hydrophobic amino acid and x represents any amino acid), which is recognized by the E2 enzyme Ubc9 [16,17,18]. However, some reports describe a large number of proteins that lack this consensus sequence but nevertheless can be SUMOylated [3]. Unfortunately, even less is known about the role of multiple SUMO modification by single SUMO moieties at different lysine residues within one target protein, or SUMO chain formation [19]. Additonally, there might be crosstalk between SUMOylation and phosphorylation [20]. 

## 2. Viral Mimicry of Ubiquitin or Ubiquitin E2/E3 Ligases 

Many viruses encode viral gene products that mimic the functions of cellular proteins, and thus interfere with key cellular processes and host cell homeostasis. Consistent with this, viruses have acquired strategies to benefit from the eukaryotic ubiquitin system. Here, we summarize the evidence that some viruses encode analogs of enzymes involved in ubiquitinylation events to mimic the conserved functions of host ubiquitin, E2/E3 enzymes, or to directly counteract E3 activity (see table 1). 

### 2.1. Viruses Expressing Viral Ubiquitin (v-ubi) Analogs 

**AcMNPV** (Autographa californica nuclear polyhedrosis virus) infects invertebrates and belongs to the family of baculoviruses with large, rod-shaped, circular DNA. This virus establishes virions, which contain many nucleocapsids within a single viral envelope. AcMNPV encodes the v-ubi protein, which has 76% identity with the eukaryotic protein, but likely retains all the functions of the human homolog [21]. Analysis of infected cells indicated that v-ubi is highly expressed during the late phase of viral infection. Several attempts to establish virus mutants depleted for v-ubi were unsuccessful. Therefore, the expression of this viral gene is essential for the viral life cycle. It is hypothesized that v-ubi might serve as a molecular chaperone for the incorporation of viral proteins into particles [21].

### 2.2. Viruses Encoding Viral Ubiquitin E2 Enzyme Counterparts 

**ASFV** (African swine fever virus) belongs to the Asfarviridae family and causes African swine fever (ASF). This large, double-stranded DNA virus with a linear genome encodes at least 150 genes and replicates in the cytoplasm of infected cells, often causing a hemorrhagic fever with high mortality rates in pigs [22]. In 1992, a study identified the first viral determinant encoded by ASFV with high homology to cellular UBC (ubiquitin-conjugating/E2) enzymes and similarities in structure and enzyme activity to the yeast ubiquitin-conjugating enzymes UBC2 and UBC3 [23]. The ASFV-encoded enzyme UBCv1 has UBC activity when expressed in *E. coli*, forming thioester bonds with ubiquitin in the presence of purified ubiquitin-activating enzyme (E1) and ATP, and subsequently transferring ubiquitin to specific protein targets. Mechanistically, the ASFV UBCv1 enzyme can form multi-ubiquitinylated conjugates of histones, di-ubiquitin, and multi-ubiquitinylated UBC/E2 enzymes and also transfers ubiquitin to histones in an E3-independent reaction [24]. 

It appears that the virus-encoded UBCv1 enzyme is involved in several steps during the virus replication cycle, such as uncoating or assembly of virus particles, transition from early to late gene expression, onset of virus DNA replication, and virus-mediated DNA repair [24]. 

Interestingly, ASFV UBCv1 has been hypothesized to affect monocyte/macrophage function by modulating ubiquitin-dependent mechanisms, although the molecular details are not known [24]. An additional role for the ASFV UBCv1 enzyme was suggested to be the manipulation of the endosome/lysosome system of monocytes or macrophages to the advantage of the virus. Thus, the acquisition of a UBC enzyme by ASFV enables the virus to exploit the host ubiquitin conjugation system, either to regulate the virus replication cycle or in some other way modify virus-host cell interactions [24].

### 2.3. Viruses Synthesizing Ubiquitin E3 Enzyme-Like Proteins 

Several reports have demonstrated that the **HAdV** (Human Adenovirus) early factors E1B-55K and E4orf6 assemble into an E3 ubiquitin ligase complex associating with host cell components Rbx1/ROC1/Hrt1, Cullin2/5, and Elongin B and C in a fashion analogous to the cellular SCF and VBC complexes, which recruit Cullin1 and Cullin2 (summarized in [25]). In the functional E3 ligase complex, E1B-55K serves as the substrate recognition unit while E4orf6 assembles the cellular components. This high molecular mass complex sequesters specific cellular substrates, such as p53, Mre11, DNA ligase IV, Integrin alpha 3, Tip60, ATRX, and SPOC1 into the ubiquitin-dependent proteasomal degradation pathway; this process is essential for HAdV replication [26,27,28,29,30,31,32,33]. 

It is also becoming increasingly apparent that HAdV possesses additional strategies to hijack cellular E3 ubiquitin ligase functions. There is evidence that E1B-55K-dependent/E4orf6-independent as well as E1B-55K-independent/E4orf6-dependent, Cullin-based E3 ubiquitin ligase activities neutralize host defense processes [34,35]. In line with this, recent work showed that HAdV also assemble E3 ubiquitin ligases in a serotype-dependent manner. These findings indicate that different HAdVs exhibit distinct Cullin specificities, and therefore target a different selection of cellular substrates [36,37]. Taken together, these findings emphasize that further detecting new host substrates during infection, as well as identifying the underlying processes by which different HAdV serotypes modulate host cell pathways, will be important for gaining further insights into how these pathways operate.

**KSHV** (Kaposi’s sarcoma-associated herpesvirus) is a gammaherpesvirus establishing lifelong infections that are usually asymptomatic in immunocompetent individuals. Like all herpesviruses, KSHV can induce latency and lytic replication. KSHV encodes two membrane-associated RING-CH (MARCH) family E3 ubiquitin ligases, K3 and K5, which direct the ubiquitinylation and subsequent internalization and endolysosomal degradation of several different plasma membrane substrates, including MHC-1 molecules [38,39,40]. K3 preferentially induces Lys63 linked poly-ubiquitinylation, which requires the E2-conjugating enzymes UbcH5b/c and Ubc13 to initiate mono- and subsequent poly-ubiquitinylation of MHC-1 prior to clathrin-dependent internalization mediated by the epsin endocytic adaptor [41]. While a positionally conserved homologue of K3 can be found in several other gammaherpesvirus genomes, the presence of more than one vMARCH gene is a unique feature of KSHV [42,43]. 

By generating a recombinant KSHV, Brulois and coworkers found that K3- and K5-dependent downregulation of MHC-1 represents stage-specific regulation of immunoevasive activity during infection [44]. Their study uncovered a role for K3 in the removal of MHC-1 from the cell surface during lytic replication. In contrast to K5, this K3 activity was restricted to the later stages of lytic replication. As for some other gammaherpesvirus genes [43,45], it has been suggested that K5 may have originated from a gene duplication event [46]. This may have facilitated the evolution of distinct roles for K3 and K5. 

**MHV-68** (Murine gammaherpesvirus 68) is closely related to human gammaherpesviruses and naturally infects mice. For MHV-68, the *in vivo* significance of the virus interfering with antigen presentation is still not clear. Deletion of the viral factor mK3 resulted in low latency during primary infection [47]. However, it did not completely abolish MHV-68-mediated downregulation of MHC-I levels relative to levels in uninfected cells. This suggests that other immunoevasins may be encoded by MHV-68 and that partial recovery of MHC-I expression is sufficient to affect latency establishment [44].

**HPV** (Human papillomaviruses) infiltrates the ubiquitin pathway through the virus-encoded E3-like enzymes E6 and E7. The HPV E6 gene product, in cooperation with the cellular E6-associated protein (E6AP), binds and induces ubiquitin-dependent degradation of cellular substrates such as p53 [48]. This is mediated by E6/E6AP acting as an ubiquitin E3 ligase in functional cooperation with the E2 conjugating enzymes UbcH5/H6/H7 [49,50]. E6/E6AP was the first viral/cellular complex with E3 enzyme capacity reported to form a ubiquitin thioester as an intermediate step prior to conjugating ubiquitin onto target proteins [51]. Beside p53 as a host substrate [52,53], many other targets are degraded by this complex, such as c-Myc, Mcm7, Bak, E6TP1, and NFX1 [54,55,56,57,58]. 

The other viral oncoprotein encoded by HPV, E7, competes for binding to the cellular tumor suppressor Rb prior to the release of the cellular E2F transcription factor to promote cell cycle progression. HPV16 E7 was shown to highjack already assembled E3 ligase containing Cullin2/Elongin B/C and Rbx1 in the infected cell to degrade Rb via the host proteasomal pathway [59,60] and to efficiently modulate substrate specificity [61]. 

**HIV** (Human immunodeficiency virus) is a lentivirus that causes AIDS, a well-known progressive failure of the immune system, by infecting CD4^+^ T cells, macrophages, and dendritic cells. The HIV RNA genome harbors several open reading frames in addition to the retroviral elements *gag*, *pol*, and *env*. Rev and Tat polypeptides are involved in viral gene regulation. Vif, Vpu, Vpr, and Nef proteins play essential roles in establishing the virus replicative environment. Among these proteins, at least three (Vif, Vpu, and Vpr) subvert cellular ubiquitin ligases to block the action of anti-viral defenses. Interestingly, investigation of mRNA synthesis revealed that the cellular cytidine deaminase APOBEC3G restricts HIV1 infectivity [62]. This is efficiently counteracted when Vif protein interacts with APOBEC3G. Vif reconstitutes an E3 ubiquitin ligase by hijacking cellular components such as Cullin5, Rbx2, and ElonginB/C [63]. To infiltrate this complex, Vif contains a conserved BC box motif for efficient ElonginB/C binding. Deletion of this BC box in Vif abolishes co-immunoprecipitation with the Elongins [64]. The HCCH zinc motif in Vif is critical for selective Cullin5 binding [65]. 

HIV Vpr is a 14 kDa virion-associated protein that promotes infection of non-dividing cells [66] and triggers G2 cell cycle arrest in dividing cells [67,68]. Vpr assembles a cellular ubiquitin ligase complex containing DCAF1**/**DDB1**/**Cullin4 [69]. Numerous targets have been discovered for this DCAF1**/**DDB1**/**Cullin4 ubiquitin ligase complex in the absence of Vpr, such as STAT1, XPC, Chk1, CDT1, Merlin, c-Jun, TSC2, and p27^kip^ [70]. However, Vpr must associate with the DCAF1**/**DDB1**/**Cullin4 ubiquitin ligase complex for Vpr-mediated G2 cell cycle arrest and DNA-damage response, macrophage infection, and Vpr-mediated degradation of the uracil-DNA glycosylases UNG2 and SMUG1 in order to increase viral fitness [71]. IRF3 represents another substrate that is degraded in the presence of Vpr [72] to support virus-mediated counteraction of host immune responses in the context of a natural infection. 

### 2.4. Viruses Blocking Host Ubiquitin E3 Enzymes by Virus-Encoded Inhibitors 

**VACV** (Vaccinia virus) belongs to the family of Poxviruses and represents large DNA viruses that replicate in the cytoplasm. VACV was the live vaccine used to eradicate smallpox [73]. Poxviruses express many immunomodulatory factors [74], such as the A49 protein, which is also conserved in other VACV strains and orthopoxviruses including variola, but not in monkeypox, camelpox, and ectromelia viruses [75]. During infection, A49 is transcribed early and late [76]. Mansur and collegues reported that A49 blocks NF-κB activation and thus contributes to virus virulence [77]. A49 exploits molecular mimicry of IκBα to bind the WD40 domain of the E3 ubiquitin ligase β-TrCP via a sequence closely related to motifs in IκBα, IκBβ, HIV Vpu, and other β-TrCP substrates. Consequently, IκBα is not ubiquitinylated or degraded and remains attached to the NF-κB complex in the cytoplasm. Hence, the VACV A49 protein mediates anti-NF-kB activity using molecular mimicry to inhibit β-TrCP E3 ubiquitin ligase function, thereby blocking NF-kB activation to promote immune evasion and enhance virus virulence.

**RV** (Rotaviruses) are double-stranded RNA viruses of the Reovirus family and are a major cause of pediatric gastroenteritis [78]. The icosahedral RV particle encapsidates a genome encoding six structural and five or six nonstructural proteins [79]. RV replicates primarily in the villous tips of the small intestine [79], where it is sensed by the RLRs (RIG-I like receptors) RIG-I and MDA5, prior to NF-κB and IRF3 activation through MAVS (mitochondrial antiviral-signaling protein). Like VACV A49 above, RV can block NF-κB activation through its nonstructural protein NSP1, which targets the E3 ubiquitin ligase β-TrCP [80,81,82]. Thus, NSP1 mimics the IκB phosphodegron to mediate degradation of β-TrCP or other immunomodulatory proteins, such as IRF3/5/7/9, MAVS, and/or TNF receptor-associated factor 2 (TRAF2) [83,84].

## 3. Virus-Mediated Cross-Talk between SUMO and Ubiquitin PTM

SIM (SUMO interacting motif)-containing SUMO-targeted ubiquitin ligases (STUbLs) mediate the cross-talk between ubiquitin and SUMO signaling by promoting the ubiquitinylation and proteasome-dependent degradation of poly-SUMOylated substrates [85]. The human RNF4 protein is the most studied STUbL [86,87] and represents the smallest ULS (ubiquitin ligases for SUMO conjugates) characterized so far. RNF4 comprises at least three SIMs, which mediate binding to SUMO-1 and SUMO-2, with a preference for chains of at least three SUMO moieties [86]. 

Recently, another STUbL protein, Arkadia/RNF111, was identified [88]. Arkadia bears at least three functional SIMs, which mediate strong binding of linear SUMO-1 or SUMO-2 chains, suggesting that the function of Arkadia involves recognizing poly-SUMO signals [88]. Taken together, the ubiquitinylation of SUMOylated proteins mainly sequesters the substrate proteins into the proteasomal pathway to control the levels of SUMOylated proteins.

**HSV1** (Human herpes simplex virus type 1) expresses the early viral protein ICP0 (Infected Cell Polypeptide 0), which is a RING finger E3 ubiquitin ligase with STUbL activity, and plays a role in efficiently initiating lytic infection as well as in reactivating latent viral genomes. HSV1 disrupts antiviral PML (promyelocytic leukemia protein) nuclear bodies in the host-cell nucleus mainly via ICP0-mediated proteasomal degradation, by targeting SUMOylated PML and Sp100 [89]. Indeed, ICP0 targets PML more efficiently than SUMO conjugates in general. Whereas PML isoforms II**–**VI require SUMOylation to be recognized by ICP0, isoform I is also targeted without SUMOylation. Upon infection with ICP0-null mutant viruses, SUMO-1 and SUMO-2/-3 conjugates are seen to accumulate [89,90,91]. 

**VZV** (Varicella zoster virus) expresses an ICP0 ortholog called ORF61p, which specifically targets the PML-NB component Sp100 and promotes dispersal of these nuclear aggregates [92]. ORF61p has three SIMs that are required for the viral factor to interact with PML nuclear bodies, whereas its additional RING domain is only required for PML-NB dispersal [93,94]. Unlike ICP0, ORF61p promotes SUMO-independent proteasomal degradation of cellular substrates such as IRF3 [95].

**MHV-68** (Murine gammaherpesvirus 68) encodes the vFGARAT protein ORF75c, which mediates proteasome-dependent degradation of the PML protein. While ORF75c does not stimulate the activity of other known regulators of PML protein stability, such as CK2 and E6AP, it possesses self-ubiquitinylation activity *in vitro* and is sufficient to increase the level of ubiquitinylated PML. These observations suggest that it may be a specific PML E3 ubiquitin ligase, especially since other known PML E3 ubiquitin ligases were not required for ORF75c-mediated PML degradation [96]. 

## 4. Viruses Hijacking De-Ubiquitinylation Processes for Efficient Replication

DUBs are major regulators in various biological processes. Thus, increasing awareness of these enzymes expressed by viruses can not only provide novel information about unknown steps in virus replication (see table 1), as well as further insights into more general aspects of this class of enzymes, but can also highlight potential sites for pharmacological intervention.

### 4.1. DNA Viruses Synthesize Viral DUBs to Counteract Ubiquitinylation Events

**HAdV** (Human Adenoviruses) encode the viral protease adenain (Avp) [97]. HAdV virions contain about 10 copies of this cysteine proteinase [98], as well as several other HAdV proteins essential for the formation of mature infectious virions [99]. DNA binding inside the virions activates the viral protease Avp to cleave 11 amino acids (pVIc) from the HAdV capsid pVI protein precursor. pVIc represents a cofactor and results in further Avp activation, which subsequently leads to proteolytic maturation of viral capsid precursor proteins inside the virion. This is a prerequisite for endosomal lysis and cytoplasmic entry of viral capsids during the initial infection cycle [100]. Activation of Avp by the viral DNA and pVIc prevents precursor protein cleavage before virion assembly, thus generating immature capsids.

In 2002, Balakirev and coworkers used a biotinylated form of a DUB inhibitor as a probe to retrieve adenain from a lysate of infected cells and observed a time-dependent increase in de-ubiquitinating activity during the late phase of infection compared to mock infected cells [101]. Moreover, *in vitro* assays with recombinant purified Avp, substrate proteins, and chemically synthesized pVIc peptides showed that Avp exhibits DUB activity against Lys48-linked poly-ubiquitin, and that the ISG15 precursor protein is a cellular substrate for this adenoviral DUB [102]. 

**Table 1:** Host cell SUMO/Ubiquitin processes infiltrated by viral mimicry.

Virus	Viral Homologue	Host Cell Function	Putative Consequence	References	
Human Adenoviruses (HAdV)	Avp	DUB	efficient viral replication	[101]	
Human Adenoviruses (HAdV)	E1B-55K E4orf6	E3 ubiquitin ligase	efficient viral replication oncogenic potential	[25]	
Human Adenoviruses (HAdV)	Avp	SENP	efficient viral replication	[103,104]	
Human Adenoviruses (HAdV)	E1B-55K	E3 SUMO ligase	p53 SUMOylation oncogenic potential	[105,106,107]	
Human Papillomaviruses (HPV)	E6	E3 ubiquitin ligase	efficient viral replication oncogenic potential	[48,49,50,51]	
Human herpes simplex virus type 1 (HSV1)	ICP0	E3 ubiquitin ligase	efficient viral replication	[89,90]	
Human herpes simplex virus type 1 (HSV1)	UL36	DUB		[108]	
Human Cytomegalovirus (HCMV)	UL36	DUB		[109]	
Kaposi’s sarcoma associated herpesvirus (KSHV)	K3 K5	E3 ubiquitin ligase	immune evasion	[38,39,40]	
Kaposi’s sarcoma associated herpesvirus (KSHV)	K-bZIP	E3 SUMO ligase		[110,111]	
Epstein-Barr virus (EBV)	BPLF1	DUB		[112]	
Murine gammaherpesvirus 68 (MHV68)	ORF75c	E3 ubiquitin ligase	efficient viral replication	[96]	
Murine gammaherpesvirus 68 (MHV68)	ORF64	DUB		[113]	
Murine gammaherpesvirus 68 (MHV68)	mK3	E3 ubiquitin ligase	immune evasion	[44,47]	
Varicella zoster virus (VZV)	ORF61p	E3 ubiquitin ligase	NF-κB inhibition	[92,93,95]	
Pseudorabies viruses (PrV)	pUL36	DUB		[114]	
African swine fever virus (ASFV)	S273R	SENP	efficient viral replication	[115]	
African swine fever virus (ASFV)	UBCv1	ubiquitin conjugating activity	uncoating/assembly early/late transition virus DNA replication virus-mediated DNA repair	[23,24]	
Vaccinia virus (VACV)	I7	SENP		[103,104]	
Vaccinia virus (VACV)	A49	E3 ubiquitin ligase inhibition	NF-κB inhibition viral virulence immune evasion	[77]	
Autographa californica nuclear polyhedrosis virus (AcMNPV)	v-ubi	ubiquitin homologue	substrate for ubiquitin processing enzymes	[21]	
Rotavirus (RV)	NSP1	E3 ubiquitin ligase inhibition	NF-κB inhibition	[80,81,82]	
Human immunodeficiency virus (HIV)	Vif	E3 ubiquitin ligase	immune evasion	[63,64,65]	
Human immunodeficiency virus (HIV)	Vpr	E3 ubiquitin ligase	immune evasion	[69]	
Crimean–Congo hemorrhagic fever virus (CCHF)	L	DUB	viral replication	[116]	
Dugbe virus (DUGV)	L	DUB	viral replication	[116]	
Nairobi sheep disease virus (NSDV)	L	DUB	viral replication	[116]	
Severe acute respiratory syndrome coronavirus (SARS CoV)	PLpro	DUB	de-ISGylation immune evasion	[117,118]	
Middle East respiratory syndrome coronavirus (MERS-CoV)	PLpro	DUB	de-ISGylation immune evasion	[117,119,120,121]	
Human coronavirus NL63 (HCoV-NL63)	PLP2	DUB	de-ISGylation immune evasion	[117,119,120,121]	
Porcine epidemic diarrhea virus (PEDV)	PLP2	DUB	de-ISGylation immune evasion	[117,119,120,121]	
Mouse hepatitis virus (MHV)	PLP2	DUB	de-ISGylation immune evasion	[117,119,120,121]	

**HSV1** (Herpes simplex virus type 1) tegument protein UL36 (VP1/2; ICP1/2) was reported to exhibit DUB activity in its N-terminal region [108]. Although the reactive sequence shows no similarity to any known DUB protein, the conservation of the Cys and His box motifs is characteristic for Cys protease DUB families [122]. The enzymatic activity of HSV1 UL36 is specific for ubiquitin, but exhibits relatively low catalytic efficiency. It was shown that UL36 disassembled Lys48 but not Lys63 poly-ubiquitin, implicating a role in protein stabilization [108]. In addition to HSV1, the presence of DUB activity with specificity for ubiquitin was also confirmed in recombinant UL36 proteins from EBV (gamma–herpesviruses), MCMV/HCMV (betaherpesviruses) [109,123], and MHV-68 (Murine gammaherpesvirus 68) ORF64 [113].

To also confirm catalytic activity in **HCMV** (Human Cytomegalovirus) extracellular particles, mutation of the respective Cys and His residues resulted in lower virus yields and delayed development of cytopathic effects. Therefore, the DUB enzymatic activity of UL36 plays a major but not essential role during HCMV replication [109]. In detail, the HCMV homologue of HSV UL36 is the UL48 protein, which is required to cleave and package the HCMV viral genome into capsids [124]. UL48 is involved in connecting incoming viral capsids to the dynein motor during transport along microtubules to the nuclear pores [125] and during retrograde axonal transport [126,127].

**EBV** (Epstein-Barr virus) is a human gammaherpesvirus that establishes latent infections and is associated with a broad spectrum of malignancies of lymphoid and epithelial cell origin. The viral protein BPLF1, the EBV homologue of HSV1 UL36 and HCMV UL48, was shown to encode viral DUB activity. Using a reporter substrate that carries ubiquitin fused to GFP, the N-terminus of BPLF1 was confirmed as a very potent ubiquitin de-conjugase. EBV BSLF1 and BXLF1 were also capable of cleaving ubiquitin [112]. These novel viral DUBs are expressed in the host nucleus during the early phase of productive EBV infection, in accordance with their reported function of the viral factors in DNA replication and nucleotide metabolism. Interestingly, the regions in BXLF1 that are essential for its DUB activity are not conserved in other herpesvirus analogs. In contrast to other herpesviruses, EBV BXLF1 was shown to localize to the centrosomes, where it encircles the tubulin-rich centrioles in a microtubule-independent manner [128]. 

**PrV** (Pseudorabies virus) belongs to the alphaherpesviruses and also encodes for the essential tegument protein pUL36 [114]. Mutational analysis of pUL36-dependent de-ubiquitinylation revealed that the inhibiting active site Cys26 led to defective virion morphogenesis accompanied by impaired neuroinvasion in a mouse infection model. Cellular or viral substrates of the cysteine protease in pUL36 are not known and, therefore, the biological role in herpesvirus replication is not understood in detail [129].

So far, all known herpesviruses encode a large tegument protein containing de-ubiquitinylating activity located within their N-terminal regions [123]. These viral DUBs function as cysteine proteases and have a strictly conserved catalytic triad comprised of Cys, His, and Asp acid residue [123]. Herpesvirus-encoded DUBs do not share sequence homology or conserved domains with known host cell DUBs [108]. It is also not completely understood whether de-ubiquitinylation of viral or cellular proteins by the UL36 DUB activity plays a part in UL36 functions, *i.e.*, nucleocapsid formation, tegumentation, budding, viral egress from and entry into host-cells, nuclear transport and entry of the herpesvirus genome. Notably, the minimal DUB consensus domain is highly conserved among human (HSV, HCMV, EBV) and murine (MCMV, MHV-68) herpesviruses and confers both hydrolytic and ubiquitin specificity [108,123].

### 4.2. DUBs Encoded by RNA Viruses Limiting Host Ubiquitinylation

**CCHF** (Crimean–Congo hemorrhagic fever virus) is the tick-borne causative agent of severe hemorrhagic fever with outbreaks and high mortality in Asia, Europe, and Africa [130,131]. These viruses have a negative-strand RNA composed of three segments designated S, M, and L [132], where the expressed L protein was shown to resemble the viral polymerase function. For CCHF and other nairoviruses, such as **DUGV** (Dugbe virus) and **NSDV** (Nairobi sheep disease virus), an ovarian tumor (OTU)-like protease motif, a predicted papain-like protease with a cysteine protease signature, could be identified located in the amino termini of the L protein [116]. L proteins are polyproteins that are autoproteolytically cleaved or involved in de-ubiquitinylation [116]. Further investigation is needed to determine what role the de-ubiquitinylation activity may play in the virus replication cycle. Intriguingly, the OTU-like protease family also includes proteins encoded by other segmented negative strand viruses (rice stripe virus) and several positive strand RNA viruses (carlaviruses, foveaviruses), which are autoproteolytically cleaved to generate the viral RNA-dependent RNA polymerase and additional proteins such as viral helicases [133,134].

**SARS CoV** (Severe acute respiratory syndrome coronavirus) is a positive and single-stranded RNA virus with an envelope surrounding the viral particle. SARS CoV is the causative agent for severe acute respiratory syndrome, which frequently leads to outbreaks and high mortality. CoV usually expresses two large polyproteins, which are cleaved into 16 smaller subunits by two coronaviral proteases, 3CLpro (3C-like protease) and PLpro (papain-like protease) [135]. A Ub-like domain was identified at the N-terminus of the PLpro catalytic core domain [136] and studies unraveling the crystal structure of SARS-CoV PLpro established high homology with the host-cell USP family of DUBs [137]. Specifically, SARS-CoV PLpro efficiently debranches Lys48 poly-ubiquitin chains and hydrolyzes general DUB substrates [117,118]. 

In addition, precursor processing activity was shown for ISG15 [118], an additional ubiquitin-like protein expressed in response to interferon induction, which is covalently conjugated to substrate proteins [138]. It is not yet known whether SARS-CoV PLpro de-ubiquitinates further substrates other than replicase polyprotein sequences *in vivo*. Since the synthesis of CoV RNA requires ongoing viral protein production, one could speculate whether de-ubiquitinylation by SARS-CoV PLpro protects replicase subunits against proteasomal degradation [139]. In addition, further studies revealed that beside SARS-CoV PLpro, **MERS-CoV** (Middle East respiratory syndrome coronavirus) PLpro; **HCoV-NL63** (Human coronavirus NL63) PLP2; **PEDV** (Porcine epidemic diarrhea virus) PLP2; and **MHV** (Mouse hepatitis virus) PLP2 also encode polypeptides with a ubiquitin-like domain located upstream of the protease domain [119], exhibiting protease, DUB, and de-ISGylating activities [117,120,121]. 

## 5. Viral Analogs of the Host SUMOylation Pathway

SUMO-1, -2, and -3, are covalently linked to their substrates via an enzymatic cascade that involves the heterodimeric activating enzyme SAE1/SAE2, the conjugating enzyme UBC9, and different classes of SUMO ligases, including the PIAS family, RING-domain-containing proteins, and members of the TRIM protein family. SUMO-specific proteases (SENPs) are required for the maturation of SUMO precursors and to reverse SUMOylation. Various viruses have evolved to take advantage of the conserved host-cell SUMOylation machinery by molecular mimicry of essential components (see table 1).

### 5.1. Viruses Mimicking Cellular SENPs

The Avp protease of **HAdV** (Human Adenoviruses) is a viral member of a new class of related cysteine proteases, which include the cellular SUMO proteases [103,104]. A crystal structure of Avp revealed that the residues implicated in catalysis can be superimposed on the corresponding active-site residues of the classical cysteine protease papain, despite the absence of primary sequence similarity between the enzymes (see below). All four key catalytic residues of the adenovirus protease are fully conserved in the cellular SENP enzymes, indicating that both protein types use a similar catalytic mechanism [103]. 

The core domain of the active site triad of Avp comprises residues His54, Glu71, and Cys122, which, in the crystal structure of this enzyme, have a spatial disposition identical to that of the catalytic triad of the classical cysteine protease papain, although the sequential order in papain is Cys25, His159, and Asn175. In addition, Gln115 is in an equivalent spatial position to the Gln19 of papain involved in forming the oxyanion hole in the active site [140]. 

Beside the adenoviral Avp protease, the **VACV** (Vaccinia virus) and **Fowlvirus** encoded I7 protease also share functional and structural similarities to the SENP proteins, which means they can be grouped in a specific kind of cystein protease family [103,104]. The I7 gene, encoding a 423-amino-acid polypeptide, is expressed late in infection [141] and harbors a C-terminal region of about 200 amino acids containing a 90 amino acid core domain that is also present in the adenovirus protease. 

**ASFV** (African swine fever virus) encodes two polyproteins, pp220 and pp62, which are cleaved to produce six major structural components of the virus particle [142]. All the proteolytic cleavages occur after the second Gly of the consensus sequence Gly-Gly-Xaa, which is also recognized as a cleavage site in the maturation of adenovirus structural proteins and in some cellular proteins [143]. The S273R gene product of ASFV was suggested to be the protease involved in processing the ASFV polyproteins. Inhibitor profiling and site-directed mutagenesis indicated a specific cysteine protease activity [115]. Studies on S273R in cells infected with ASFV support a role for this protein in the processing of the viral polyproteins at late times of infection, when S273R is localized within the cytoplasmic viral factories where morphogenesis occurs. S273R is also present in the core of mature virus particles [115]. Whether the ASFV protease also cleaves other viral or even cellular substrates early in infection is a question that remains to be addressed.

### 5.2. Viruses Expressing SUMO Ligase-Like Activity

The **HAdV** (Human Adenoviruses) early viral protein E1B-55K was reported to possess E3 SUMO ligase functions that specifically target p53 when both factors are relocalized into PML nuclear bodies [105,106,107]. Since p53 is one of the most recognized regulators of cell cycle arrest and apoptosis, E1B-55K-induced PTM of p53 with SUMO proteins represents a key event in the oncogenic transformation of primary cells induced by adenoviral oncoproteins [105]. E1B-55K is itself a substrate for the host cell SUMO modification system, harboring a classical SCM (SUMO conjugation motif) around lysine 104 [20,144,145]. Indeed, PTM of E1B-55K is known to be involved in several aspects of viral protein function, such as functional inactivation of the tumor suppressor protein p53, proteasomal degradation of the chromatin remodeling factor Daxx, as well as oncogenic potential. 

The K-bZIP protein encoded by **KSHV** (Kaposi’s sarcoma-associated herpes virus) is another viral protein that utilizes the SUMO pathway to alter the host cell cycle. K-bZIP is expressed after acute infection or reactivation of the latent genome and represents the structural analogue of the EBV transcription factor Zta. Interestingly, K-bZIP appears to be modified by all three SUMO isoforms. Like the adenoviral E1B-55K, K-bZIP is a strong transcriptional repressor whose activity depends on efficient SUMO conjugation [110,146]. It has been proposed that K-bZip functions as a viral SUMO ligase with a preferential selectivity towards SUMO-2/-3 paralogs, allowing recruitment of Ubc9 to KSHV promoter sites followed by transcriptional repression [111]. K-bZip induces SUMO modification of itself as well as on interaction partners such as p53 and pRB. Consistently, K-bZip is recruited to p53 target sites, dependent on the viral protein’s SIM, and affects multiple p53 downstream genes in a SUMO-dependent manner [111]. 

## 6. Conclusions

Viruses utilize host ubiquitinylation and SUMOylation processes to enhance viral replication and evade cellular immune responses. These host enzymatic cascades are involved in all physiological processes, and thus influence the balance between normal and pathogenic cellular signaling and determine the final outcome of each viral infection. Beside orchestrating PTMs on cellular and viral proteins, viruses have evolved several strategies to infiltrate such cascades, including molecular mimicry of key molecules such as ubiquitin, ubiquitin ligases, and ubiquitin and SUMO de-conjugating enzymes (see table 1). Ultimately, this modulates the host cell homeostasis and supports enhanced replication or limits an effective host response to prevent virus elimination.

Soon after the discovery of ubiquitin and SUMO, these enzymatic processes were suggested to be connected with virus pathogenesis. However, many molecular events are still not understood in detail. Some 25 years ago, ubiquitin-like genes were discovered in viral genomes that inactivate the ubiquitin-dependent antiviral defense of the host cell by acting as ubiquitin analogs [21,147]. Indeed, manipulation of the ubiquitinylation and SUMOylation systems are emerging as a key theme in viral pathogenesis, and examples of viral proteins reported to mimic or redirect such activities in order to modify the cellular environment in favor of virus persistence or efficient replication are accumulating.

Usurping ubiquitin/SUMO pathways was recently also demonstrated in proteins encoded by some pathogenic bacteria, such as SseL, a Salmonella de-ubiquitinase required for macrophage killing, and Yersinia factor YopJ, which acts as a de-ubiquitinase to inhibit NF-κB activation [148,149,150]. Together these findings imply that such enzymes play general roles in regulating pathogenic infections. However, the systematic identification of viral analogs for ubiquitin or SUMO enzymes is a complicated task due to the wide sequence variation of the known candidates. Moreover, viral proteins are often quite different in sequence and domain organization from their mammalian counterparts, hampering the identification of viral analogs.

Despite the difficulties involved and future work required, the systematic identification of viral factors that interfere with the ubiquitin/SUMO machineries will be rewarding since it will provide novel insights into unknown virus/host interactions and unravel important features of viral pathogenesis. Moreover, the information presented here highlights that ubiquitin/SUMO pathways are promising targets for development of specific antiviral therapeutic interventions. 
